# Phosphorylation dynamics of sodium-independent organic anion transporter SLCO4A1 and its role in diseases: a review

**DOI:** 10.3389/fsysb.2026.1780024

**Published:** 2026-07-31

**Authors:** Jaytha Thomas, Suhail Subair, Althaf Mahin, Athira Perunelly Gopalakrishnan, Prathik Basthikoppa Shivamurthy, Athira C. Rajeev, Rajesh Raju

**Affiliations:** Centre for Integrative Omics Data Science, Yenepoya (Deemed to be University), Mangalore, Karnataka-, India

**Keywords:** cancer, disease, organic anion transporter, phosphorylation, SLCO4A1

## Abstract

SLCO4A1, a membrane-localized organic anion transporter belonging to the solute carrier (SLC) family, mediates the sodium-independent transport of endogenous substrates, including steroid hormones, prostaglandins, and thyroid hormones. Despite increasing evidence supporting its physiological and pathological significance, a comprehensive review focusing on its phosphorylation landscape and phosphoregulation is currently lacking. Large-scale phosphoproteomic analyses have identified three predominant phosphorylation sites, T37, S40, and S43, within its N-terminal intrinsically disordered region, suggesting that phosphorylation is a potential regulator of SLCO4A1 localization, stability, and substrate specificity. Predicted upstream kinases, including MAPKs, CDKs, PAKs, and HIPK2, further link SLCO4A1 to signaling pathways governing cellular stress responses, inflammation, and cell-cycle progression. Aberrant expression of both SLCO4A1 and its antisense long non-coding RNA, SLCO4A1-AS1, has been reported in multiple malignancies, including colorectal, ovarian, lung, thyroid, and gastric cancers, where they are associated with poor clinical outcomes and activation of oncogenic signaling pathways. Beyond cancer, SLCO4A1 has also been implicated in inflammatory vascular disorders such as Behçet’s disease and Kawasaki disease. This review comprehensively summarizes the structural organization, phosphoregulation, and disease relevance of SLCO4A1, with particular emphasis on phosphorylation as a central determinant of its transport activity and signaling functions.

## 1 Introduction

The solute carrier (SLC) superfamily is essential for modulating cellular metabolism and signaling, as it enables the movement of diverse substances, such as cellular compounds, environmental toxins, therapeutic agents, small molecules, metabolites, hormones, trace elements, and xenobiotics, across plasma membranes into and out of cells (Alam et al., 2023). This superfamily includes 66 families and approximately 430–450 members, categorized by sequence homology and functional roles (Ferrada and Superti-Furga, 2022). Within this group, organic anion-transporting polypeptides (OATPs), encoded by *SLCO* genes, are sodium-independent carriers that facilitate the uptake of organic anions into cells, including bile acids, conjugated steroids, thyroid hormones, and numerous pharmaceuticals. Humans possess 11 OATPs, grouped into six families (OATP1 through OATP6), where the OATP4 family consists of OATP4A1 and OATP4C1 (Obaidat et al., 2012). OATP4A1, produced by the *SLCO4A1* gene, is alternatively known as the sodium-independent organic anion transporter E, OATP-E, or simply OATP4A1.

The *SLCO4A1* gene resides on chromosome 20q13.33 [HGNC] (https://www.genenames.org/) and directs the synthesis of a 12-transmembrane domain protein comprising roughly 722 amino acids, predominantly anchored at the plasma membrane (Buxhofer-Ausch et al., 2020). Its primary sites of expression include the liver, placenta, and heart (Obaidat et al., 2012). SLCO4A1 transports a range of native compounds, such as conjugated steroid hormones (e.g., estrone-3-sulfate), prostaglandins (especially PGE2), and thyroid hormones (T3 and T4), as well as foreign chemicals and medications, operating primarily as an organic anion exchanger (Koller et al., 2022). In addition to transport, SLCO4A1 participates in multiple cancer-related and signaling cascades, affecting processes like cell growth, motility, invasiveness, and inflammatory responses (Li S. et al., 2024). Nonetheless, alterations in SLCO4A1 levels can perturb localized signaling systems, potentially contributing to pathological conditions (Koller et al., 2022).

Interest in SLCO4A1 has surged owing to evidence of its dysregulated expression across various malignancies. For instance, in colorectal cancer, Ban and colleagues observed upregulation of SLCO4A1 in roughly 32% of tumor specimens, positioning it as a possible prognostic marker tied to disease advancement and spread (Ban et al., 2017). In high-grade serous ovarian cancer (HGSOC), SLCO4A1 levels varied but were often elevated, with increased expression aligning with heightened activity in inflammatory gene programs, such as those involving NF-κB, NOD-like receptors, TNFR2, and CD40 signaling (Koller et al., 2022). In non-small cell lung cancer (NSCLC), SLCO4A1 emerges as a candidate prognostic indicator; it is overexpressed in NSCLC tissues and cell lines, where it drives cellular proliferation, migration, and invasion (Li S. et al., 2024). Collectively, these observations indicate that SLCO4A1 functions as a pro-tumorigenic transporter, influencing metabolic activities and immune-related pathways.

Moreover, the SLCO4A1 genomic region encodes SLCO4A1-AS1, an antisense long non-coding RNA that regulates signalling cascades and gene activity. Aberrant SLCO4A1-AS1 expression has been noted in multiple cancers, such as colorectal and lung types, implying its involvement in tumor initiation and its value as a prognostic tool (Yu et al., 2018; Tang et al., 2019; Chen Y. L. et al., 2023). At a mechanistic level, SLCO4A1-AS1 exerts effects by producing microRNAs with tumor-suppressing properties, binding to proteins like TOX4, and possibly influencing *SLCO4A1* gene transcription (Chen Y. L. et al., 2023).

Despite advances in understanding SLCO4A1, important gaps remain, particularly regarding its phosphorylation dynamics and potential coregulatory networks, which could be elucidated through analysis of existing mass spectrometry data. Furthermore, the therapeutic implications of targeting SLCO4A1 or its antisense RNA, SLCO4A1-AS1, are still unclear. Addressing these uncertainties is critical given the role of transporters like SLCO4A1 in modulating drug sensitivity and influencing tumour-immune interactions. This review consolidates current knowledge of SLCO4A1’s structural characteristics, phosphorylation landscape and functional role in diseases, highlighting its potential as both a diagnostic biomarker and a therapeutic target.

## 2 Sequence and molecular characteristics of SLCO4A1

**FIGURE 1:**
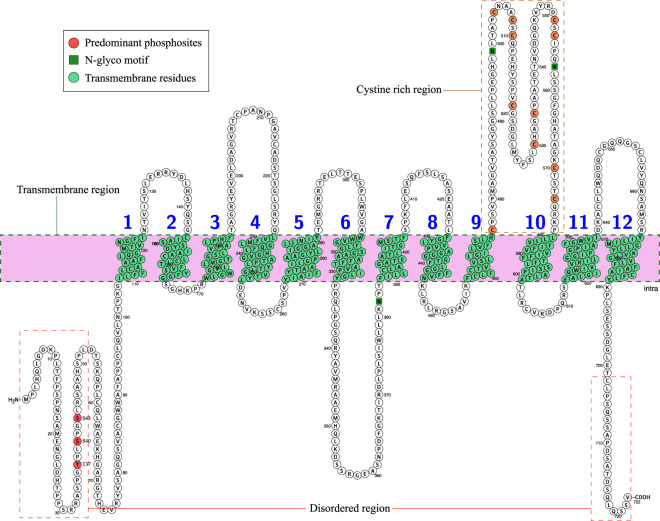
Linear schematic representation of the SLCO4A1 protein topology. SLCO4A1 comprises 12 predicted transmembrane helices, a cysteine-rich extracellular loop between TM9 and TM10, and predicted intrinsically disordered N-terminal (residues 1–52) and C-terminal (residues 702–722) regions. Conserved N-glycosylation motifs are shown in green, predominant phosphorylation sites (T37, S40, and S43) in red, and transmembrane residues within the membrane bilayer.

The *SLCO4A1* gene encodes a protein of 722 amino acids that functions as an organic anion transporter (Hagenbuch, 2007). The protein exhibits a predicted molecular weight of approximately 80 kDa, though earlier reports described an apparent mass of ∼65 kDa under certain experimental conditions (Hagenbuch, 2007). As a member of the OATP family, SLCO4A1 possesses 12 anticipated transmembrane α-helices (Tamai et al., 2000) (Figure 1). A prominent extracellular loop between the ninth and tenth transmembrane helices contains ten conserved cysteine residues that are essential for proper trafficking to the plasma membrane and likely form disulfide bridges (Tonduru et al., 2022). The conserved OATP signature sequence, D-X-RW-(I/V)-GAWW-X-G-(F/L)-L, is located at the boundary between the third extracellular loop and the sixth transmembrane domain. Transmembrane domains 1–10 play a critical role in substrate recognition and binding (Buxhofer-Ausch et al., 2020). N-linked glycosylation occurs at multiple asparagine residues on the second and fifth extracellular loops, contributing to protein stability and transport efficiency (Yao et al., 2012).

The N-terminal region (amino acids 1–52) and C-terminal tail (amino acids 703–722) are predicted to be intrinsically disordered regions (IDRs) [InterPro] (https://www.ebi.ac.uk/interpro/). These flexible segments lack a fixed three-dimensional structure and can adopt multiple conformations, facilitating interactions with kinases and other regulatory proteins and thereby conferring additional regulatory versatility [(Moses et al., 2023; Modic et al., 2024)].

SLCO4A1 functions as a sodium-independent facilitative transporter mediating cellular uptake of a wide range of organic anions (Li S. et al., 2024). Established substrates include estrone-3-sulfate, dehydroepiandrosterone sulfate, thyroxine (T4), triiodothyronine (T3), reverse T3, and prostaglandin E_2_ (PGE_2_) (Wang et al., 2021; Tamai et al., 2000). In the human placenta, SLCO4A1 is predominantly expressed on the apical membrane of syncytiotrophoblasts, where it plays a key role in extracting maternal thyroid hormones from the maternal circulation to support fetal brain development and metabolic regulation (Loubière et al., 2010; Hagenbuch, 2007; Sato et al., 2003). Variations in SLCO4A1 expression or activity may therefore influence prostaglandin-mediated inflammation, steroid hormone homeostasis, and thyroid hormone balance across multiple tissues (Koller et al., 2022). To date, no experimental three-dimensional structure of SLCO4A1 has been determined.

## 3 Phosphorylation landscape and predominant phosphorylation sites of SLCO4A1

Phosphorylation events targeting the N-terminal intrinsically disordered region (IDR) of SLCO4A1 point to a pivotal post-translational control mechanism that could fine-tune its transport capabilities (Newcombe et al., 2022; Usher et al., 2024). IDRs constitute pliable protein domains devoid of a stable three-dimensional architecture, which permits diverse structural rearrangements and positions them as pivotal nodes in intracellular communication networks (Trivedi and Nagarajaram, 2022). Within transmembrane proteins, phosphorylation in these flexible zones critically governs aspects such as folding, structural integrity, intracellular relocation, and intermolecular associations (Kjaergaard and Kragelund, 2017).

**FIGURE 2:**
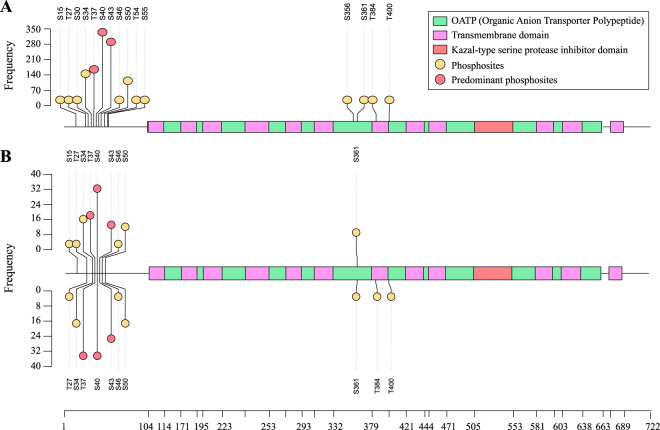
Lollipop plot illustrating the frequency distribution of SLCO4A1 phosphorylation sites from phosphoproteome profiling and differential regulation datasets. **(A)** Class 1 phosphorylation sites detected in SLCO4A1 across profiling datasets. **(B)** Class 1 phosphorylation sites showing differential regulation in SLCO4A1 across quantitative differential datasets.

A substantial number of phosphorylation sites on SLCO4A1 have been documented in the literature across a range of biological and experimental contexts, posing difficulties in their systematic compilation and documentation. To mitigate this and ensure the robustness of our analysis, the present review focused exclusively on high-confidence phosphorylation sites derived from investigations involving human cells. Criteria for inclusion required a localization probability of 75% or higher, or an ambiguity score exceeding 13. The assembled datasets were divided into two categories: (1) those contrasting specific biological or experimental conditions against appropriate controls, and (2) those documenting phosphorylation sites observed within defined biological or experimental contexts. Differential abundance of class-1 phosphosites was determined using straightforward thresholds, including a fold change of ≥1.3 for upregulation or ≤0.76 for downregulation, alongside a p-value <0.05 to establish statistical relevance. For uniformity, all proteins were aligned to their canonical HGNC (https://www.genenames.org/) gene symbols, and phosphorylation sites were aligned to corresponding UniProt entries via a bespoke computational pipeline (Raghu et al., 2025; Sheela et al., 2026). After evaluating over 3,825 accessible global phosphoproteomics datasets from human cells, 376 profiling datasets and 120 differential datasets were found to contain class-1 phosphosites within SLCO4A1-derived phosphopeptides. This analysis revealed a total of 20 distinct SLCO4A1 phosphosites in the profiling datasets and 12 in the differential ones. Closer examination of the compiled data highlighted three sites as the most observed: pS40, pS43, and pT37, which are all localized within the flexible N-terminal intrinsically disordered region (amino acids 1–52), implying their significance as primary sites for regulatory modulation (Figure 2).

## 4 Predicted upstream kinases regulating SLCO4A1

Multiple investigations and database repositories have aided in pinpointing potential upstream kinases involved in modulating SLCO4A1 phosphorylation. Well-established phosphorylation repositories, including PhosphoSitePlus (Hornbeck et al., 2015), PhosphoELM (Dinkel et al., 2011) and RegPhos (Huang et al., 2014), have documented experimentally confirmed upstream kinases alongside their associated phosphosites. Complementing this experimental data, predictive computational tools like NetworKIN (Linding et al., 2008), AKID (Parca et al., 2019), and iKiP-DB (Mari et al., 2022; Fahma et al., 2025) have been employed in contemporary research to forecast kinase-substrate interactions through motif sequence analysis and network-derived associations. Furthermore, expansive phosphoproteomic compilations, such as those from synthetic peptide-based assays by Johnson et al. (2023) and the functional phosphosite inventory by Ochoa et al. (2020), have broadened insights into kinase engagements that may govern SLCO4A1 activity.

**FIGURE 3:**
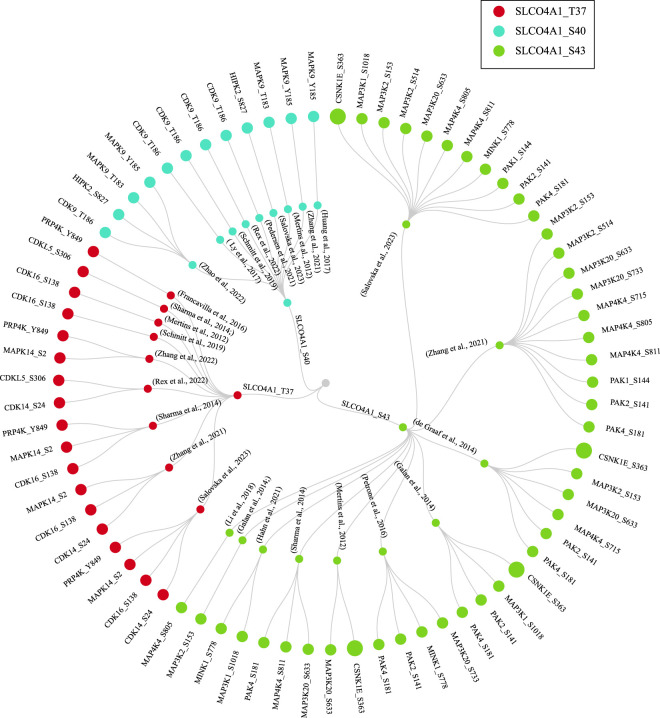
Predicted upstream kinases identified among the coregulated proteins of SLCO4A1, along with the curated source.

Our phosphoproteomic findings indicate that a variety of kinases target distinct phosphorylation loci to control SLCO4A1 function. For the S40 phosphosite on SLCO4A1 (Figure 3), four prospective upstream kinases have been implicated, including mitogen-activated protein kinase 9 (MAPK9) (Y185, T183), cyclin-dependent kinase 9 (CDK9) (T186), and homeodomain-interacting protein kinase 2 (HIPK2) (S827), according to predictions from Johnson et al. (2023). The adjacent pS43 site engages a broader kinase repertoire, encompassing mitogen-activated protein kinase kinase kinase 20 (MAP3K20) (S633, S733), mitogen-activated protein kinase kinase kinase kinase 4 (MAP4K4) (S811, S805, S715), misshapen-like kinase 1 (MINK1) (S778), mitogen-activated protein kinase kinase kinase 2 (MAP3K2) (S153, S514), casein kinase I isoform epsilon (CSNK1E) (S363), and mitogen-activated protein kinase kinase kinase 1 (MAP3K1) (S1018), as forecasted by Johnson et al. (2023). Predictions from platforms like NetworKIN and AKID have additionally nominated upstream kinases for pS40, such as serine/threonine-protein kinase PAK 4 (PAK4) (S181), casein kinase I isoform epsilon (CSNK1E) (S363), serine/threonine-protein kinase PAK 2 (PAK2) (S141), and serine/threonine-protein kinase PAK 1 (PAK1) (S144). In addition, the T37 site is connected to five forecasted upstream kinases they are cyclin-dependent kinase 14 (CDK14) (S24), mitogen-activated protein kinase 14 (MAPK14) (S2), cyclin-dependent kinase-like 5 (CDKL5) (S306), cyclin-dependent kinase 16 (CDK16) (S138), and serine/threonine-protein kinase PRP4 homolog (PRP4K) (Y849), as detailed by Johnson et al. These associations underscore the intricate, kinase-orchestrated governance of SLCO4A.

## 5 Other kinases among the coregulated network of SLCO4A1

To explore the signalling context of SLCO4A1, we analysed the full set of kinases using curated phosphorylation resources such as PhosphoSitePlus, PhosphoELM, and RegPhos. In addition to these experimental datasets, we incorporated predictions from computational tools including NetworKIN, AKID, and iKiP-DB, which identified kinases showing coordinated phosphorylation changes within the same dataset. The SLCO4A1-associated kinase network reveals extensive crosstalk with pathways that could potentially modulate transporter expression, membrane trafficking, substrate specificity, or downstream cellular responses.

**FIGURE 4:**
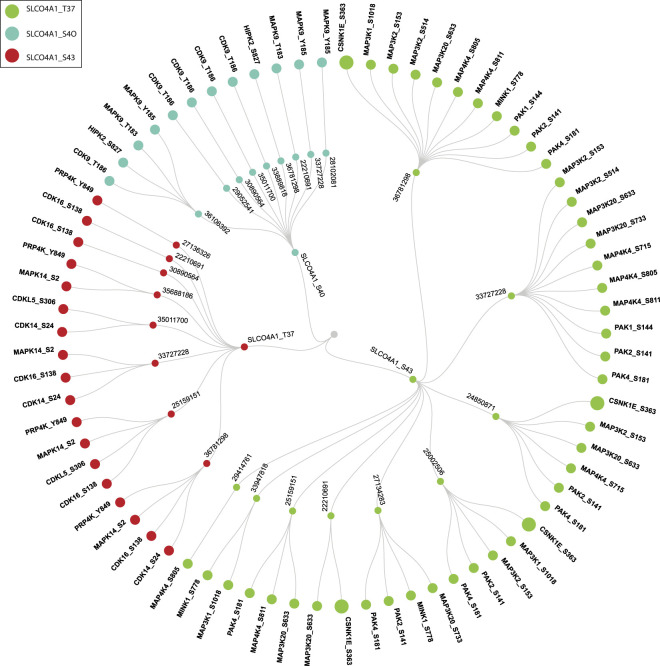
Catalogued list of other kinases and their families identified among the coregulated network of SLCO4A1.

In total, 181 kinases were detected (Figure 4). Prominent families and examples include MAPK cascade and related kinases are Mitogen-activated protein kinase kinase kinase 1 (MAP3K1), Mitogen-activated protein kinase kinase kinase 7 (MAP3K7), Mitogen-activated protein kinase kinase kinase 2 (MAP3K2), Mitogen-activated protein kinase kinase kinase 11 (MAP3K11), Mitogen-activated protein kinase kinase kinase 20 (MAP3K20), Mitogen-activated protein kinase kinase kinase kinase 3 (MAP4K3), Mitogen-activated protein kinase kinase kinase kinase 4 (MAP4K4), CDK family, Cyclin-dependent kinase 9 (CDK9) (T186), Cyclin-dependent kinase 16 (CDK16) (S95, S138), Cyclin-dependent kinase 13 (CDK13) (T1246, S358), Cyclin-dependent kinase 11A/B (CDK11A/B) (T583, S752, S763), Cyclin-dependent kinase 14 (CDK14) (S24), Cyclin-dependent kinase-like 5 (CDKL5) (S306). AGC family, Ribosomal protein S6 kinase beta-2 (RPS6KB2) (S416, S221), Ribosomal protein S6 kinase alpha-1 (RPS6KA1) (S221), Ribosomal protein S6 kinase alpha-3 (RPS6KA3) (S17), Ribosomal protein S6 kinase delta-1 (RPS6KC1) (S560), RAC-alpha serine/threonine-protein kinase (AKT1) (T308, S124), RAC-beta serine/threonine-protein kinase (AKT2) (S474), cAMP-dependent protein kinase type II-alpha regulatory subunit (PRKAR2A) (T54), Protein kinase C epsilon type (PRKCE) (S329), Serine/threonine-protein kinase LATS1 (LATS1) (T612), CMGC group MAPK9/JNK2 (T183, Y185), Glycogen synthase kinase-3 beta (GSK3B) (S9), Homeodomain-interacting protein kinase 2 (HIPK2) (S827). STEfamily, Serine/threonine-protein kinase 3 (STK3) (S385), Mitogen-activated protein kinase kinase kinase 1 (MAP3K1) (S292), Mitogen-activated protein kinase kinase kinase 7 (MAP3K7) (S455), Mitogen-activated protein kinase kinase kinase 2 (MAP3K2) (S514), Mitogen-activated protein kinase kinase kinase kinase 3 (MAP4K3) (S329). The CAMK family are Serine/threonine-protein kinase MARK2 (MARK2) (S569), 5′-AMP-activated protein kinase catalytic subunit alpha-1 (PRKAA1/AMPKα1) (S508), Calcium/calmodulin-dependent protein kinase type 1 (CAMK1) (S363), Serine/threonine-protein kinase Chk2 (CHEK2) (S120) and related Casein kinase I isoform alpha (CSNK1A1) (T323), Tau-tubulin kinase 2 (TTBK2) (S786).

This broad kinase repertoire indicates that SLCO4A1 operates within a highly interconnected, multilayered phosphosignalling environment involving serine/threonine and tyrosine kinases from virtually all major branches of the human kinome. While direct upstream regulation of SLCO4A1 by these kinases remains to be experimentally validated, their co-regulation strongly suggests that SLCO4A1 activity, localisation, or downstream effects can be modulated by multiple signalling pathways known to govern cell proliferation, stress responses, inflammation, and membrane transporter dynamics-pathways frequently rewired in cancer and other pathologies.

## 6 Phosphatases among the coregulated network of SLCO4A1

**FIGURE 5:**
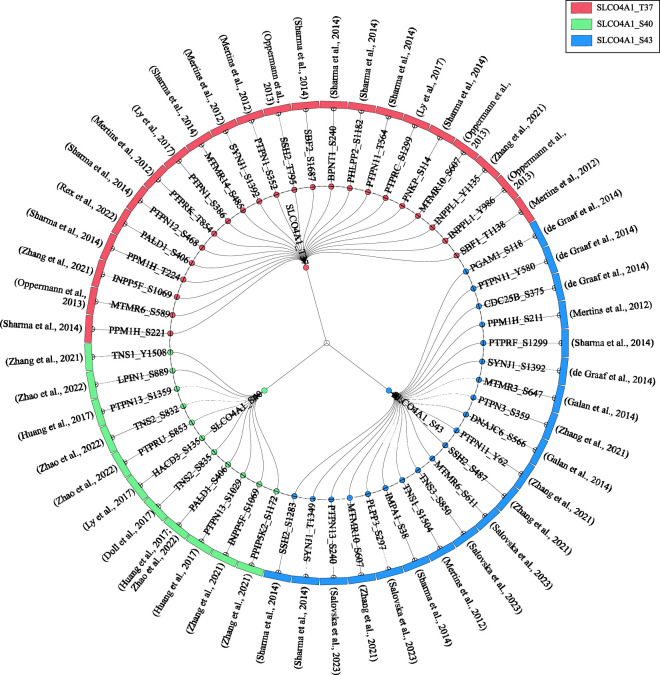
Phosphatases identified among the coregulated proteins of SLCO4A1, along with the curated source.

Analysis of the SLCO4A1-associated phosphoproteomic network, a collection of 54 phosphatases co-regulated with SLCO4A1 was identified using specialised dephosphorylation resources, including the Dephosphorylation Database and the DesPho (DepPho) tool (Figure 5). The majority belong to the protein tyrosine phosphatase (PTP) superfamily, with prominent examples including Receptor-type tyrosine-protein phosphatase alpha (PTPRA) (S189), Tyrosine-protein phosphatase non-receptor type 13 (PTPN13) (S1029, S1359, S240), Receptor-type tyrosine-protein phosphatase U (PTPRU) (S853), Tyrosine-protein phosphatase non-receptor type 11 (PTPN11/SHP2) (Y580, Y62, T564), Receptor-type tyrosine-protein phosphatase F (PTPRF) (S1299), Receptor-type tyrosine-protein phosphatase C (PTPRC/CD45) (S1299), Tyrosine-protein phosphatase non-receptor type 3 (PTPN3) (S359), Tyrosine-protein phosphatase non-receptor type 12 (PTPN12) (S468), Tyrosine-protein phosphatase non-receptor type 1 (PTPN1) (S386, S352), Receptor-type tyrosine-protein phosphatase kappa (PTPRK) (T854). These tyrosine-directed phosphatases highlight extensive tyrosine-based regulatory control within the network. Additionally, several members of the myotubularin family of lipid phosphatases were detected Myotubularin-related protein 10 (MTMR10) (S607), Myotubularin-related protein 14 (MTMR14) (S485), Myotubularin-related protein 3 (MTMR3) (S647), Myotubularin-related protein 6 (MTMR6) (S611, S589). These enzymes primarily dephosphorylate phosphatidylinositol 3-phosphate (PI3P) and phosphatidylinositol 3,5-bisphosphate (PI(3,5)P_2_), thereby modulating endosomal trafficking, membrane dynamics, and phosphoinositide-dependent signalling.

Collectively, the enrichment of both protein tyrosine phosphatases and myotubularin-related lipid phosphatases indicates that SLCO4A1 function is integrated into a sophisticated dephosphorylation network governing tyrosine signalling, phosphoinositide homeostasis, cytoskeletal remodelling, vesicle trafficking, and plasma membrane organisation, processes critical for transporter localisation, activity, and response to extracellular cues.

## 7 Role of SLCO4A1 in diseases

While SLCO4A1 is primarily physiological in healthy tissues, its dysregulation, particularly overexpression, has been implicated in various pathological conditions, most notably cancers and inflammatory diseases. No major monogenic disorders are directly caused by SLCO4A1 mutations, but associations exist with conditions like primary hypertrophic osteoarthropathy and cystadenocarcinoma.

### 7.1 SLCO4A1 in cancer

Emerging evidence positions SLCO4A1 as an oncogenic factor in multiple malignancies, often correlating with tumor progression, poor prognosis, and altered cellular behaviors such as proliferation, migration, invasion, and resistance to therapy.

In colorectal cancer, it is overexpressed in 32%–40% of cases, directly drives proliferation, colony formation, migration, and invasion, and independently predicts poor overall and disease-free survival (Ban et al., 2017; Zhang et al., 2022). In non-small cell lung cancer (including LUAD), it fuels tumour growth and EMT via sustained MAPK/ERK activation, upregulates Ki-67 and PCNA, and confers radioresistance through crosstalk with STC2, TERT, CYP24A1, and ALDH3A1 (Li S. et al., 2024; Wang et al., 2025). In HPV-positive cervical cancer, HPV16 E6/E7 oncoproteins directly transactivate SLCO4A1, which then cooperates with MST1R (RON kinase), ACAN, and nicotinic receptors to drive malignant transformation (Renteria-Flores et al., 2025). High-grade serous ovarian cancer shows strong SLCO4A1 enrichment in inflammation-related pathways (NOD-like receptor, NF-κB, TNFR2, adipocytokine, CD40) and markedly worse survival (Koller et al., 2022), while in papillary thyroid cancer, it independently predicts poor progression-free survival and links to neutrophil-mediated immunity (Wang et al., 2021). In meningioma, SLCO4A1 correlates with immune infiltration by NK cells, macrophages, and neutrophils, highlighting its role in tumour–immune crosstalk (Chen et al., 2023a) (Table 1).

**TABLE 1:** Role of SLCO4A1 and SLCO4A1-AS1 in human cancers.

Gene	Cancer type	Key findings/Function	Key pathways/Interacting molecules	PMID
SLCO4A1	Colorectal Cancer	Overexpressed in 32% of cases (n = 84); predicts poor OS; knockdown decreases proliferation, migration, invasion, colony formation	​	Ban et al. (2017)
SLCO4A1	Non-Small Cell Lung Cancer (NSCLC)	Upregulated; poor OS; promotes proliferation & EMT	MAPK, Ki-67, PCNA, vimentin, E-cadherin	Li et al. (2024b)
SLCO4A1	High-Grade Serous Ovarian Cancer	High expression; linked to inflammation pathways; poor OS	NOD-like, adipocytokine, TALL1, CD40, NF-κB, TNFR2	Koller et al. (2022)
SLCO4A1	Papillary Thyroid Cancer	High expression; independent predictor of poor PFS; neutrophil immunity	Neutrophil-mediated immunity	Wang et al. (2021)
SLCO4A1	Cervical Cancer (HPV-related)	Upregulated by HPV16 E6/E7; contributes to transformation	MST1R, ACAN, CHRNA3	Renteria-Flores et al. (2025)
SLCO4A1	Colon Adenocarcinoma (COAD)	Overexpressed; poor prognosis; negative correlation with anti-tumour immune infiltrates	CD8^+^ T cells, B cells, dendritic cells	Chen et al. (2023c)
SLCO4A1	Meningioma	Potential therapeutic target linked to immune infiltration	NK cells, macrophages, neutrophils	Chen et al. (2023a)
SLCO4A1	Breast Cancer (computational)	Predicted target of glycyrrhizin with anti-cancer potential	​	Poustforoosh et al. (2024)
SLCO4A1-AS1	Melanoma	Upregulated; knockdown decreases proliferation/migration/invasion, increases apoptosis	miR-1306-5p - > PCGF2	Wang et al. (2022)
SLCO4A1-AS1	Non-Small Cell Lung Cancer (NSCLC)	Upregulated; promotes progression; potential biomarker	miR-223-3p - > IKKα/NF-κB	Li et al. (2020)
SLCO4A1-AS1	Glioblastoma	Promotes proliferation and invasion	SOX2, super-enhancers	Wu et al. (2025)
SLCO4A1-AS1	colorectal cancer	Facilitates growth and metastasis	β-catenin-dependent Wnt pathway	Yu et al. (2018)
SLCO4A1-AS1	Hepatocellular Carcinoma (HCC)	Involved in immune-excluded microenvironment changes	SLCO family	Luo et al. (2025)
SLCO4A1-AS1	Lung Cancer Metastasis (context-specific)	Suppresses invasion/migration (rare tumour-suppressor behaviour)	TOX4 - > NTSR1 axis	Chen et al. (2023b)

The antisense lncRNA SLCO4A1-AS1 acts as a powerful oncogenic amplifier and context-specific modulator. In colorectal cancer, promoter hypomethylation drives its overexpression; it then functions as a molecular scaffold for Hsp90-Cdk2 interaction, stabilises Cdk2, phosphorylates c-Myc at Ser62, and accelerates G1-S transition (Zhang et al., 2022). In gastric cancer, melanoma, and NSCLC, it operates as a classical ceRNA, sequestering miR-149-5p, miR-1306-5p, and miR-223-3p to de-repress STAT3, PCGF2 (a Polycomb repressive complex component), and IKKα/NF-κB, respectively - collectively promoting proliferation, migration, invasion, and apoptosis resistance (Li et al., 2021; Wang et al., 2022; Li et al., 2020). In glioblastoma, SLCO4A1-AS1 sustains SOX2 expression via super-enhancer regulation, maintaining stemness and invasiveness (Wu et al., 2025). In digestive system cancers, it activates canonical Wnt signalling to enhance motility (Li P. et al., 2024), while in hepatocellular carcinoma, it participates in immune-excluded microenvironment remodelling (Luo et al., 2025). Interestingly, in certain lung cancer metastatic contexts, SLCO4A1-AS1 suppresses invasion via the TOX4–NTSR1 axis, revealing rare context-dependent tumour-suppressive behaviour (Chen et al., 2023b).

Emerging computational and pharmacological evidence further broadens its relevance in breast cancer, SLCO4A1 is predicted as a high-confidence target of the natural compound glycyrrhizin (Poustforoosh et al., 2024), and in colon adenocarcinoma it negatively correlates with antitumour immune infiltrates (CD8^+^ T cells, B cells, dendritic cells), suggesting a role in immune evasion (Chen et al., 2023c).

In summary, the SLCO4A1/SLCO4A1-AS1 bidirectional locus represents a rare pan-cancer oncogenic module that integrates multiple hallmarks of cancer sustained proliferative signalling (MAPK, c-Myc, Wnt), replicative immortality (TERT), resisting cell death, EMT and metastasis, genome instability (super-enhancers), inflammation, and immune escape. Its recurrent dysregulation, through genetic, epigenetic, viral, and microenvironmental mechanisms, and its direct druggability as a transporter make this axis one of the most promising emerging targets for diagnostics, prognostication, and therapy across colorectal, lung, ovarian, cervical, thyroid, brain, gastric, melanoma, liver, and breast cancers.

### 7.2 SLCO4A1 in non-cancer diseases

Beyond its well-established oncogenic role, SLCO4A1 and its regulatory lncRNA are increasingly implicated in a broad spectrum of non-neoplastic inflammatory, immune, and degenerative disorders, highlighting the transporter’s pivotal function in cellular stress responses, drug/xenobiotic handling, and immune homeostasis (Table 2). In acute lung injury, SLCO4A1 mediates uptake of the natural flavonoid lysionotin, thereby activating AMPK/Nrf2 signalling and conferring potent antioxidant and lung-protective effects (Xiong et al., 2025). Genetic variants in the SLCO4A1 locus, particularly lncRNA-Slco4a1 polymorphisms, are significantly associated with susceptibility to Kawasaki disease (Amirsardari et al., 2025) and ocular complications in Behçet’s disease, mapping near the MICA/HLA region (Hatemi et al., 2024; Casares-Marfil et al., 2023). During SARS-CoV-2 infection, SLCO4A1 is markedly upregulated as part of a disrupted drug-metabolising enzyme and transporter (DMET) gene signature, suggesting its potential as a biomarker of altered pharmacokinetics in severe COVID-19 (Nwabufo et al., 2024). In neurodegenerative conditions, SLCO4A1 participates in ceRNA networks that modulate neuroinflammation in Parkinson’s disease miR-411/1193/301b axis (Chun and Kim, 2024) and is overexpressed in Alzheimer’s disease as an oxidative stress hub gene alongside NFKBIA and CLIC1 (Hu et al., 2023). In idiopathic pulmonary fibrosis, it emerges as a diagnostic hub linked to neutrophil-driven inflammation (Lin et al., 2023), whereas in ischemic cardiomyopathy it is downregulated and serves as an immune-related diagnostic biomarker (Zheng et al., 2023). Even in veterinary immunology, alternative splicing of SLCO4A1 correlates with differential immune responses in calves (Li et al., 2023). Collectively, these findings position SLCO4A1 not merely as a cancer-associated transporter but as a versatile, context-dependent modulator of inflammation, oxidative stress, drug uptake, and immune regulation across acute lung injury, autoimmune vasculitides, viral infections, neurodegeneration, fibrosis, and cardiovascular disease, making it an attractive candidate for repurposing existing OATP inhibitors or developing novel modulators in non-oncological settings.

**TABLE 2:** Involvement of SLCO4A1 in non-cancer inflammatory, immune, and degenerative diseases.

Disease/Condition	SLCO4A1 function/Role	Pathways/Interacting molecules	PMID
Acute Lung Injury/ARDS	Uptakes Lysionotin - activates AMPK/Nrf2; antioxidant protection	AMPK, Nrf2	Xiong et al. (2025)
Kawasaki Disease	Genetic polymorphisms (lncRNA-Slco4a1) linked to susceptibility	lncRNA variants	Amirsardari et al. (2025)
Behçet’s Disease (ocular involvement)	SLCO4A1 SNPs correlate with ocular lesions	MICA/HLA region	Hatemi et al. (2024), Casares-Marfil et al. (2023)
COVID-19	Upregulated in SARS-CoV-2; biomarker in drug-metabolism disruption	DMET genes	Nwabufo et al. (2024)
Parkinson’s Disease	Part of ceRNA network regulating neuroinflammation	miR-411/1193/301b	Chun and Kim (2024)
Alzheimer’s Disease	Upregulated; oxidative-stress-related gene	NFKBIA, CLIC1 (module partners)	Hu et al. (2023)
Idiopathic Pulmonary Fibrosis (IPF)	Diagnostic hub gene linked to neutrophils	Inflammation/immune infiltration	Lin et al. (2023)
Ischemic Cardiomyopathy (ICM)	Downregulated immune-related diagnostic biomarker	Immune response genes	Zheng et al. (2023)
Immune Response in Calves (Agri-biological)	Alternative splicing linked to immune differences	AKR1C4, immunity pathways	Li et al. (2023)

## 8 Therapeutic and prognostic implications

Currently, highly selective pharmacological inhibitors of SLCO4A1 are lacking. While broad-spectrum OATP inhibitors such as rifampicin, cyclosporine A, and certain tyrosine kinase inhibitors can block SLCO4A1-mediated transport in preclinical assays, their poor selectivity often results in off-target effects on other OATP family members (Marie et al., 2017). No SLCO4A1-specific small molecules have progressed to clinical development, and antibody-based strategies, including monoclonal antibodies or antibody-drug conjugates, remain unexplored for this target. Preclinical studies have used non-selective OATP inhibition to modulate drug uptake or mitigate toxicity in cancer and inflammatory models (Marie et al., 2017). Still, the therapeutic effects of specific SLCO4A1 blockade have yet to be rigorously tested *in vivo*.

More broadly, current pharmacological approaches to SLC transporters largely rely on such broad-spectrum inhibitors rather than target-specific agents. Examples include the SGLT2 inhibitors canagliflozin (Nomura et al., 2010) and dapagliflozin (Meng et al., 2008) (SLC5A2), tiagabine (Meldrum and Chapman, 1999) (SLC6A1), atomoxetine (Ramoz et al., 2009) (SLC6A2), methylphenidate (Kasparbauer et al., 2015) (SLC6A3), and probenecid (García-Rodríguez et al., 2023) (SLC22 family), AZD3965 (SLC16A1) (Wright and Lee, 2022) and NBMPR (SLC29A1) (Wright and Lee, 2022), with additional SLC-targeting agents currently under clinical development for other family members. Consequently, the absence of SLCO4A1-specific inhibitors represents a significant therapeutic gap within this broader landscape and highlights the need for the development of selective pharmacological agents that can accurately assess SLCO4A1’s therapeutic potential without broadly disrupting physiological transporter function.

From a prognostic perspective, elevated SLCO4A1 (or its antisense lncRNA SLCO4A1-AS1) expression is associated with poor clinical outcomes in colorectal cancer (Yu et al., 2018), bladder cancer (Yang et al., 2019), gastric cancer (Fang et al., 2021), and glioblastoma (Wu et al., 2025). In non-small cell lung cancer, the picture is mixed: SLCO4A1-AS1 has been reported as tumor-promoting in one study (Li et al., 2020) and as tumor-suppressive in another (Goodman et al., 1999), suggesting its role is context- or subtype-dependent rather than uniformly oncogenic. Notably, SLCO4A1-AS1 knockdown has been shown to suppress proliferation, invasion, and tumor growth in xenograft models in colorectal cancer (Yu et al., 2018), bladder cancer (Yang et al., 2019), and gastric cancer (Fang et al., 2021).

**FIGURE 6:**
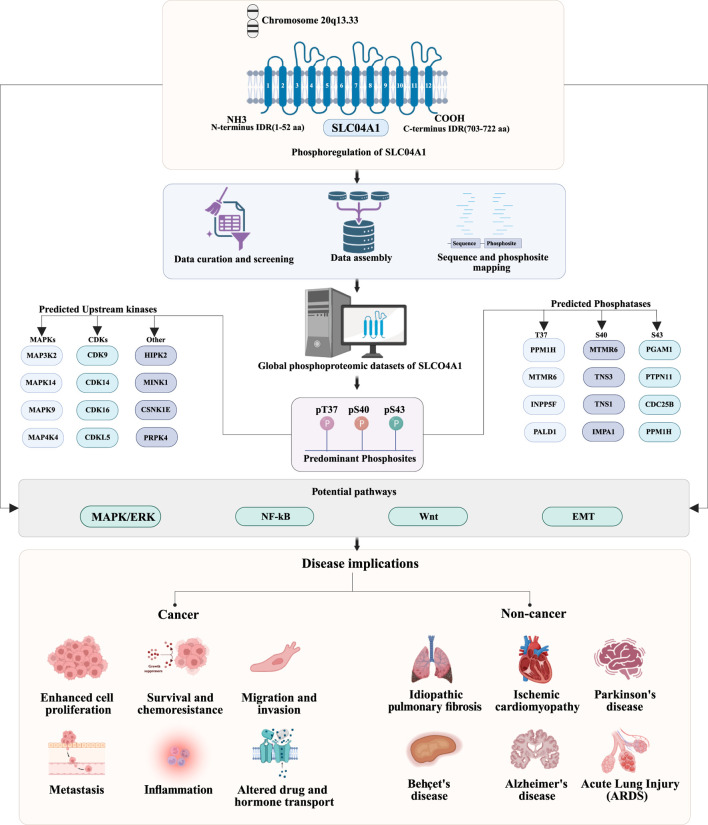
Visual summary of the phosphoregulatory landscape of SLCO4A1. The figure illustrates the protein structure, phosphoproteomic data integration workflow, identification of predominant phosphorylation sites (pT37, pS40, and pS43), predicted upstream kinases and phosphatases, associated signaling pathways, and the potential roles of SLCO4A1 dysregulation in cancer and non-cancer diseases.

In summary, although SLCO4A1 plays important physiological roles in normal tissues, its overexpression, frequently driven by the lncRNA SLCO4A1-AS1, is linked to oncogenesis and adverse prognosis in several gastrointestinal cancers and glioblastoma, with a more context-dependent role in lung cancer. Additional research is required to elucidate substrate-specific mechanisms and develop precision-targeted, SLCO4A1-selective interventions. A visual summary of this review article is provided as Figure 6.

## 9 Limitations of the study

While this review synthesizes a comprehensive picture of SLCO4A1 phosphorylation dynamics based on large-scale phosphoproteomic datasets and predictive kinase-substrate modeling, several important limitations must be acknowledged. All phosphorylation sites (including the predominant N-terminal residues T37, S40, and S43) and the associated upstream kinases (such as MAPKs, CDKs, PAKs, HIPK2, and others) discussed herein are derived exclusively from computational predictions, motif analyses, and correlative phosphoproteomic studies across diverse human cell types and conditions. To date, none of these predicted regulatory events have been directly and functionally validated in the specific context of SLCO4A1 biology.

Furthermore, no site-directed mutagenesis studies (e.g., phosphodeficient mutants such as T37A, S40A, or S43A, or phosphomimetic variants) have been reported for SLCO4A1. Similarly, targeted pharmacological investigations using kinase inhibitors or activators to modulate these pathways and assess consequent changes in SLCO4A1 activity are lacking. This gap limits our ability to establish causal relationships between specific phosphorylation events and phenotypic outcomes.

Additional challenges arise when considering SLCO4A1 as a potential therapeutic target. Its broad expression in critical tissues such as the liver, placenta, heart, and other organs, combined with its essential role as a sodium-independent transporter for key endogenous molecules, including thyroid hormones (T3 and T4), prostaglandins (e.g., PGE_2_), and conjugated steroids, raises significant hurdles for drug development. Inhibiting or modulating SLCO4A1 could lead to on-target toxicities or contraindications, such as disruption of thyroid hormone homeostasis, altered prostaglandin-mediated inflammatory responses, or impaired handling of other xenobiotics and drugs that rely on this transporter. Off-target effects on related OATP family members may further complicate selectivity.

These factors highlight the need for highly tissue- or context-specific targeting strategies (e.g., via antibody-drug conjugates, nanoparticle delivery, or isoform-selective modulators) and thorough preclinical safety profiling. Addressing these limitations and challenges through integrated functional studies, advanced disease models, and careful medicinal chemistry optimization will be essential to determine whether the oncogenic and inflammatory roles of SLCO4A1 can be safely exploited therapeutically. In summary, direct experimental testing of the proposed phospho-regulatory model, ideally through a combination of site-directed mutagenesis, functional transport assays, and targeted kinase modulation, remains a critical priority for future research.

## 10 Conclusion

SLCO4A1, a sodium-independent organic anion-transporting polypeptide, has emerged as a multifaceted regulator of cellular homeostasis whose dysfunction contributes to a broad spectrum of human diseases. Its canonical role in the uptake of endogenous substrates, such as thyroid hormones, prostaglandins, and steroid conjugates, is tightly regulated by post-translational phosphorylation, predominantly within the intrinsically disordered N-terminal region. Large-scale phosphoproteomic evidence consistently identifies T37, S40, and S43 as dominant regulatory sites, placing SLCO4A1 under the control of an extensive kinome network that includes MAPKs, CDKs, PAKs, HIPK2, and numerous other serine/threonine kinases linked to stress, cell-cycle, and inflammatory signaling. This intricate phosphoregulatory layer likely modulates transporter localisation, stability, substrate specificity, and non-transport signaling functions, although direct experimental validation of individual kinase–substrate relationships remains a priority for future work.

Emerging evidence suggests that dysregulation of the bidirectional *SLCO4A1/SLCO4A1-AS1* locus contributes to oncogenic processes across multiple cancer types. Overexpression of SLCO4A1, frequently amplified by its antisense lncRNA through diverse mechanisms (promoter hypomethylation, ceRNA activity, super-enhancer regulation, and viral transactivation), sustains proliferative signaling, epithelial–mesenchymal transition, radioresistance, chronic inflammation, and immune evasion across colorectal, lung, ovarian, thyroid, cervical, gastric, brain, and other malignancies. The recurrent association with unfavourable prognosis and therapy resistance underscores its clinical relevance as both a prognostic biomarker and a potential therapeutic target.

Beyond oncology, SLCO4A1 participates in inflammatory and immune homeostasis, with documented roles in acute lung injury, vasculitides (Kawasaki and Behçet’s diseases), viral infection (SARS-CoV-2), neurodegeneration, fibrosis, and cardiovascular disorders. These findings highlight the transporter’s context-dependent versatility and its integration into pathways governing oxidative stress, drug/xenobiotic handling, and immune regulation.

In conclusion, SLCO4A1 exemplifies how a single membrane transporter, through sophisticated phosphorylation-dependent regulation and bidirectional transcriptional control by its antisense lncRNA, can orchestrate diverse pathological processes ranging from oncogenesis to sterile and infectious inflammation. Its druggable nature as an OATP family member, combined with the tractability of SLCO4A1-AS1 for nucleic acid-based or small-molecule interventions, positions this axis as one of the most promising emerging targets for precision diagnostics and therapy across oncology and inflammatory diseases. Future research should prioritise functional dissection of key phosphorylation events, elucidation of substrate-specific oncogenic mechanisms, and preclinical evaluation of SLCO4A1/SLCO4A1-AS1-directed therapeutic strategies.
